# Association of triglyceride-glucose index and its related parameters with functional disability: evidence from the China Health and Retirement Longitudinal Study

**DOI:** 10.3389/fnagi.2025.1488124

**Published:** 2025-05-19

**Authors:** ZhiHong Wei, GuanHua Nie, Ji Li, Jiali Zhang, HaoLin Liu, MinXuan Ma

**Affiliations:** ^1^1Hospital-Acquired Infection Control Department, Affiliated Hospital of Jiangsu University, Zhenjiang, China; ^2^Department of Nursing, Baoying People’s Hospital, Yangzhou, China; ^3^Kunming Municipal Hospital of Traditional Chinese Medicine, The Third Affiliated Hospital of Yunnan University of Chinese Medicine, Kunming, China; ^4^Institute for Experimental and Clinical Pharmacology and Toxicology, Center of Brain, Behavior and Metabolism, University of Lübeck, Lübeck, Germany; ^5^No.965 Hospital, Joint Logistics Support Force of Chinese PLA, Jilin, China

**Keywords:** ADL limited, ADL-IADL limited, TyG, TyG-BMI index, TyG-WC index, TyG-WHtR index

## Abstract

**Background:**

The triglyceride-glucose (TyG) index, a marker of insulin resistance, is linked to mortality in coronary artery disease, ischemic stroke, and heart failure. However, its association with functional disability remains unclear. This study explored the relationship between the TyG index, its related parameters, and functional disability.

**Methods:**

Data were obtained from the China Health and Retirement Longitudinal Study (CHARLS). Functional disability was evaluated using the Activities of Daily Living (ADL) and Instrumental Activities of Daily Living (IADL) scales. Multivariate logistic regression and restricted cubic spline (RCS) models were used to assess the associations between TyG-related indices and the risk of functional disability. Subgroup analyses stratified by age, sex, smoking, drinking, and diabetes were performed. Additionally, propensity score matching (PSM) was applied to ensure the robustness of the findings.

**Results:**

The diagnosis rates were 1,510(16.32%) for ADL limited and 2,620(28.32%) for ADL-IADL limited. The TyG-related indices were significantly associated with ADL limited. [TyG index: Full adjusted OR = 1.23, 95%CI: 1.16–1.30, *p* < 0.001; TyG-BMI index: Full adjusted OR = 1.11, 95%CI: 1.05–1.18, *p* < 0.001; TyG-WC index: Full adjusted OR = 1.20, 95%CI: 1.13–1.27, *p* < 0.001; TyG-WHtR index: Full adjusted OR = 1.07, 95%CI: 1.01–1.14, *p* = 0.025]. The TyG-related indices were significantly associated with ADL-IADL limited. [TyG index: Full adjusted OR = 1.20, 95%CI: 1.14–1.26, *p* < 0.001; TyG-BMI index: Full adjusted OR = 1.13, 95%CI: 1.08–1.19, *p* < 0.001; TyG-WC index: Full adjusted OR = 1.14, 95%CI: 1.09–1.20, *p* < 0.001; TyG-WHtR index: Full adjusted OR = 1.13, 95%CI: 1.07–1.18, *p* < 0.001].

**Conclusion:**

Our findings indicated a trend that, TyG-related indices significantly associated with increased risks of ADL limited and ADL-IADL limited populations.

## 1 Introduction

Population aging is a critical societal challenge that has attracted global attention. Functional impairment, a significant concern for the health and well-being of the elderly, is characterized by limitations in physical or mental capabilities, mobility issues, and cognitive or sensory deficits. The assessment of functional impairment is commonly conducted through two established scales: Activities of Daily Living (ADL) and Instrumental Activities of Daily Living (IADL) (Katz et al., 1970; Spector et al., 1987; Lawton and Brody, 1969). ADL measures the ability to perform routine daily tasks independently, while IADL evaluates the capacity to carry out more complex tasks necessary for independent living within a community setting (Kingston et al., 2018).

Recent studies have highlighted the role of inflammation, immune responses, and chronic infections in the etiology of functional decline (Aiello et al., 2008). Furthermore, muscle functional impairment has been implicated as a key factor leading to limitations in ADL (Oida et al., 2003). A study published in 2023 found that sarcopenia, characterized by a significant loss of muscle mass and strength, is strongly associated with an increased risk of ADL dependency among older adults (Kim and Choi, 2013). It has been shown in recent studies that higher TyG index values are associated with a greater likelihood of developing mobility limitations and cognitive decline in older populations (Wang et al., 2022). Notably, emerging research points to the triglyceride-glucose (TyG) index as a potential predictor of frailty (Yuan et al., 2023). The TyG index, serves as a convenient and reliable surrogate for insulin resistance (IR) (Wang et al., 2021). By integrating fasting triglyceride and glucose measurements, the TyG index provides a valuable assessment of an individual’s insulin sensitivity. Consequently, the TyG index may offer an early warning of impending functional impairment, particularly among middle-aged and older adults.

Traditional obesity-related measures, such as body mass index (BMI) and waist circumference (WC), have been widely used to assess metabolic risk but do not fully capture the complex metabolic alterations associated with functional decline. To enhance predictive accuracy, the TyG index has been combined with obesity indices, including TyG-BMI, TyG-WC, and TyG-WHtR (waist-to-height ratio). However, despite their potential, the associations between TyG-related indices and functional impairment remain underexplored, particularly in aging populations. In this study, we aim to elucidate these relationships by utilizing baseline data from the 2011 CHARLS, focusing on the correlation between the TyG index and its combined obesity indices with respect to functional impairment. The integration of TyG with obesity indices may offer a novel perspective on the complex interplay between metabolic health and physical functionality in older adults.

## 2 Materials and methods

### 2.1 Study design and population

This study presents a secondary analysis of data sourced from CHARLS, a comprehensive national initiative that focuses on the collection of policy and health-related data from the adult population aged 45 years and above. CHARLS is specifically designed to address the multifaceted challenges associated with an aging population. Initiated in 2011, the first wave of the survey encompassed a diverse sample of participants from both rural and urban regions, employing a multistage, stratified, and proportional-to-size sampling methodology to ensure representativeness. The methodology and participant characteristics of CHARLS have been extensively documented in prior publications (Zhao et al., 2014). The dataset has become a valuable resource in the field of epidemiological research. The study protocol for CHARLS was granted approval by the Institutional Review Board of Peking University, with the reference number IRB00001052-11015.

**Figure 1:**
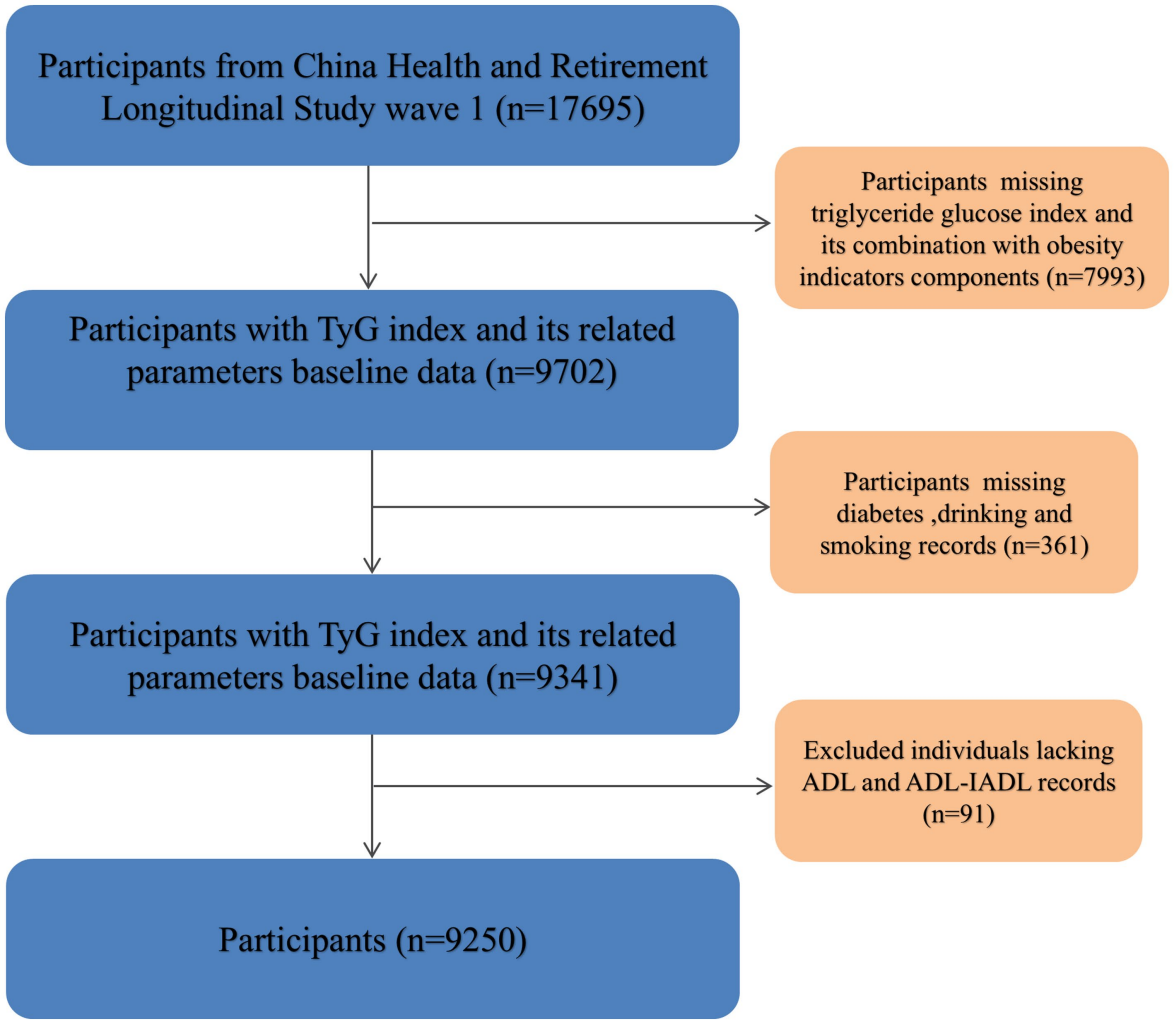
Flow-chart of participant selection process.

In accordance with the previously delineated study protocol, the inaugural survey phase conducted between 2011 and 2012 was established as the baseline for our analysis (wave 1). Participants with incomplete data were systematically excluded from the study. Specifically, individuals without recorded height measurements (*n* = 4,100), weight data (*n* = 61), waist circumference (*n* = 39), or triglyceride and glucose levels (*n* = 3,793), diabetes (*n* = 109),drinking and smoking (*n* = 252) were omitted. Additionally, participants lacking records pertaining to ADL and IADL (*n* = 91) were also excluded to ensure the integrity of the analysis. Following these exclusions, a final cohort of 9,250 participants was ascertained for inclusion in the subsequent analysis, as depicted in Figure 1.

### 2.2 TyG index and its related parameters

Plasma glucose (FPG) and triglyceride levels were assessed utilizing a Hitachi 7,180 chemistry analyzer, a reliable instrument manufactured by Hitachi in Tokyo, Japan. The precision of the blood marker measurements was ensured, with a coefficient of variation (CV) of less than 5%, indicating high reproducibility and accuracy of the analytical results. Anthropometric measurements, including height, weight, and waist circumference (WC), were conducted three times to minimize variability, and the mean of these measurements was utilized for the final data analysis. Body mass index (BMI) was derived using the standard formula: weight in kilograms divided by the square of height in meters (kg/m^2^). The waist-to-height ratio (WHtR) was determined by dividing waist circumference by height. The TyG index, a marker of insulin resistance, was calculated using the formula: Ln [triglycerides (mg/dL) × glucose (mg/dL)/2]. To enhance the predictive power of the TyG index, it was combined by incorporating BMI, WC, and WHtR, resulting in the TyG-BMI, TyG-WC, and TyG-WHtR indices, respectively.

TyG-BMI = Ln [triglycerides (mg/dL) × glucose (mg/ dL)/2] × BMI (kg/m^2^), and TyG-WC = Ln [triglycerides (mg/dL) × glucose (mg/ dL)/2] × WC (cm). TyG-WHtR = Ln [triglycerides (mg/dL) × glucose (mg/dL)/2] × waist (cm)/Height (cm). These related indices aim to provide a more comprehensive assessment of metabolic health in relation to body composition and distribution.

### 2.3 The define of ADL or ADL-IADL

Activities of Daily Living (ADL) pertain to the fundamental physical capabilities necessary for carrying out routine tasks such as dressing, bathing, self-feeding, transferring from bed to chair, toileting, and continence. Instrumental Activities of Daily Living (IADL) extend beyond basic ADL and involve more complex activities associated with independent living, including housekeeping, meal preparation, shopping, financial management, and medication adherence (Kingston et al., 2018).

Participants were surveyed on their ability to perform these activities, with the question, “Do you experience any difficulty with these activities due to physical, psychological, emotional, or cognitive reasons?” The response scale ranged from (1) do it without difficulty, (2) do it but with difficulty, (3) do it with difficulty and need help, (4) cannot do it. The responses were aggregated to produce a composite score, with the ADL score ranging from 0 to 6 and the IADL score from 0 to 5.

ADL and IADL were divided into two categories: unlimited ADL/IADL (the first options were selected in every item) and limited ADL and IADL (the last three options were chosen in any of the items). Consistent with prior research, binary variables were employed to categorize participants: those with no difficulty versus those with one or more difficulties. Specifically, an ADL limitation was identified by the presence of difficulties in one or more ADL tasks, while ADL-IADL disability was defined by difficulties in one or more tasks across both ADL and IADL domains (Zhong et al., 2017).

### 2.4 Inclusion of covariates

Data on sociodemographic characteristics and health-related factors were systematically collected using baseline questionnaires. The sociodemographic variables encompassed age, gender, education level categorized as primary school and below, middle school, and high school and above, marital status classified as married or other, and residential status indicating rural or urban residence. Health-related factors included assessments of smoking and alcohol consumption, with responses recorded as yes or no. Smoking status was categorized as “never smoked” or “smoker,” which included both “current smokers” and “former smokers.” Similarly, alcohol status was defined as “never drank alcohol” or “drinker,” encompassing both “current drinkers” and “former drinkers.” Hypertension was diagnosed based on systolic blood pressure readings of 140 mmHg or higher, diastolic blood pressure readings of 90 mmHg or higher, or self-reported history of hypertension diagnosis coupled with the current use of antihypertensive medication. Diabetes was identified through fasting blood glucose levels of 7.0 mmol/L or higher, self-reported history of diabetes diagnosis, or the use of any antidiabetic medication. Dyslipidemia was defined by total cholesterol levels of 240 mg/dL or higher, low-density lipoprotein levels of 160 mg/dL or higher, triglyceride levels of 200 mg/dL or higher, self-reported history of hyperlipidemia diagnosis, or the use of any lipid-lowering medication.

### 2.5 Statistical analysis

Baseline characteristics of continuous variables exhibiting normal distribution were reported as mean ± standard deviation (SD), whereas non-normally distributed continuous variables were presented as median (interquartile range, IQR). Categorical variables were depicted in terms of frequencies and percentages. These baseline characteristics were categorized based on whether there were limitations in ADL alone or in both ADL and ADL-IADL. A binary logistic regression analysis was conducted to estimate the ORs and their corresponding 95% CIs for the association between the TyG-related indices and the presence of limitations in ADL and ADL-IADL. Additionally, the study explored the relationship between quartiles of the TyG index and its associated parameters with the presence of ADL and ADL-IADL limitations. We normalize the tyg correlation index. The linear trends across quartiles of TyG, TyG-WC, TyG-WHtR, and TyG-BMI were examined by treating the median values within each quartile as a continuous variable in the regression model. The analysis was adjusted for potential confounders in two models: Model 1 served as the unadjusted model, while Model 2 included adjustments for age and gender. Model 3 further incorporated adjustments for residence, marital status, education level, smoking history, alcohol history, and the history of hypertension, dyslipidemia, and diabetes.

To delineate the dose–response relationships, whether linear or non-linear, between the TyG index and its related indices – TyG-WC, TyG-WHtR, and TyG-BMI and the limitations in ADL and ADL-IADL, we employed restricted cubic spline analyses. These analyses were conducted at the 5th, 50th, and 95th percentiles of the respective distributions of the TyG indices. To control for the influence of extreme values, three knots were strategically placed, excluding the most extreme 5% of the data points. Furthermore, subgroup analyses were conducted within specific demographic and lifestyle subgroups of the study population, categorized by age, gender, smoking status, alcohol consumption, and history of diabetes. These analyses aimed to scrutinize the associations between the TyG-related indices and the presence of ADL and ADL-IADL limitations within these defined subgroups.

To further explore the potential impacts of interaction effects, sensitivity analyses were conducted: (1) analysis excluding patients aged >80 years; (2) propensity score matching; (3) Adjusted for physical activity and white blood cell count based on Model 3. (4) Patients with a BMI greater than 40 were excluded.

All statistical analyses were performed using R software (version 4.1.2; R Foundation for Statistical Computing, Vienna, Austria), and a two-sided *p* value < 0.05 was considered statistically significant.

## 3 Results

### 3.1 Baseline characteristics of the participants

From the CHARLS database, a total of 9,250 participants with complete records for ADL and ADL-IADL, as well as relevant laboratory data, were identified. In the study, the group without limitations in ADL consisted of 7,740 cases, while the group with ADL limitations included 1,510 cases. Furthermore, the group without restrictions in both ADL and IADL accounted for 6,630 cases, and the group with limitations in ADL-IADL comprised 2,620 cases. The prevalence of ADL limitations was 16.32% (1,510/9,250), while the prevalence of ADL-IADL limitations in the entire sample was 28.32% (2,620/9,250). The clinical characteristics distinguishing the groups with and without ADL limitations, as well as ADL-IADL limitations, are detailed in Table 1. Individuals with ADL limitations, as compared to those without, were significantly older, predominantly male, more frequently married, and more likely to reside in rural areas. They also exhibited lower levels of education and a higher prevalence of comorbidities, including hypertension, diabetes, and dyslipidemia. Furthermore, they demonstrated elevated indices of TyG, TyG-BMI, and TyG-WC. However, no significant differences were observed between the two groups regarding TyG-WHtR, smoking status, or alcohol consumption history.

**Table 1:** Baseline characteristics of participants with ADL limited and ADL-IADL limited.

Characteristics	ADL unlimited (*n* = 7,740)	ADL limited (*n* = 1,510)	*p*	ADL-IADL unlimited (*n* = 6,630)	ADL-IADL limited (*n* = 2,620)	*p*
Age, mean ± SD	58.32 ± 9.45	62.74 ± 9.77	<0.001	59.01 ± 9.68	59.11 ± 9.55	0.626
Gender, male (%)	4,085 (52.78)	881 (58.34)	<0.001	3,521 (53.11)	1,445 (55.17)	0.073
Marriage, *n* (%)	6,851 (88.51)	1,263 (83.64)	<0.001	5,821 (87.80)	2,292 (87.51)	0.708
Rural, *n* (%)	4,959 (64.07)	1,093 (72.38)	<0.001	4,354 (65.67)	1,698 (64.83)	0.446
Drinking, *n* (%)	3,011 (38.93)	567 (37.57)	0.324	2,548 (38.46)	1,029 (39.30)	0.453
Smoking, *n* (%)	3,038 (39.26)	575 (38.08)	0.391	2,631 (39.68)	981 (37.47)	0.050
AGE, age ≥ 60 (%)	3,233 (41.77)	930 (61.59)	<0.001	2,964 (44.71)	1,199 (45.78)	0.349
Educational, *n* (%)			<0.001			0.418
Primary and below	5,267 (68.1)	1,242 (82.25)		4,669 (70.46)	1840 (70.28)	
Junior secondary school	1,650 (21.33)	200 (13.25)		1,323 (19.97)	527 (20.13)	
High school and above	817 (10.56)	68 (4.50)		634 (9.57)	251 (9.59)	
TyG, M (Q₁, Q₃)	8.59 (8.22, 9.02)	8.67 (8.28, 9.20)	<0.001	8.58 (8.21, 9.01)	8.67 (8.28, 9.14)	<0.001
TyG-BMI, M (Q₁, Q₃)	198.71 (175.53, 227.12)	202.52 (176.01, 235.66)	<0.001	198.07 (175.15, 226.60)	203.31 (177.09, 232.95)	<0.001
TyG-WC, M (Q₁, Q₃)	725.30 (652.27, 810.92)	748.95 (666.30, 847.67)	<0.001	726.27 (652.41, 810.78)	737.14 (661.12, 830.46)	<0.001
TyG-WhtR, M (Q₁, Q₃)	3.14 (2.77, 3.60)	3.15 (2.74, 3.71)	0.292	3.13 (2.76, 3.58)	3.20 (2.79, 3.68)	<0.001
Hypertension, *n* (%)	1894 (24.60)	509 (33.93)	<0.001	1715 (26.00)	688 (26.45)	0.658
Diabetes, *n* (%)	409 (5.33)	142 (9.54)	<0.001	369 (5.62)	182 (7.02)	0.011
Dyslipidemia, *n* (%)	692 (9.12)	181 (12.29)	<0.001	618 (9.52)	254 (9.89)	0.594

ADL, activity of daily living; IADL, instrumental activity of daily living; TyG, triglyceride and glucose; BMI, body mass index; WC, waist circumference; WhtR, waist-to-height ratio; SD, standard deviation; M, median; Q1, 1st quartile; Q3, 3rd quartile.

When comparing the ADL-IADL Unlimited group with the ADL-IADL Limited group, the latter was more likely to have a history of diabetes and exhibited higher indices across all TyG-related measures, including TyG, TyG-BMI, TyG-WC, and TyG-WHtR. Notably, there were no discernible differences between these groups in terms of age, gender, marital status, place of residence, education level, or the prevalence of hypertension or dyslipidemia.

### 3.2 Associations of TyG index and its related parameters with ADL limited

Table 2 delineates the statistical associations between the TyG index and its respective indices—TyG-BMI, TyG-WC, and TyG-WHtR—with ADL Limited. Utilizing logistic regression model 2, adjusted for age and sex, we observed a positive correlation between increased TyG index values and the likelihood of ADL Limited. This association retained its statistical significance following further adjustments for a comprehensive set of covariates, including residence, marital status, education level, smoking habits, alcohol consumption, hypertension, dyslipidemia, and diabetes, as detailed in model 3. The adjusted OR for the association between the TyG index and ADL Limited were 1.23 (95% CI: 1.16–1.30, *p* < 0.001), indicating a strong and significant relationship. For the TyG-BMI index, the adjusted OR was 1.11 (95% CI: 1.05–1.18, *p* < 0.001), also demonstrating a significant association, as did the TyG-WC index with an adjusted OR of 1.20 (95% CI: 1.13–1.27, *p* < 0.001). The TyG-WHtR index showed a slightly higher adjusted OR of 1.07 (95% CI: 1.01–1.14, *p* = 0.025), which was likewise statistically significant.

**Table 2:** Associations of TyG and its related parameters with ADL limited.

Characteristics	Model 1		Model 2		Model 3	
	OR (95%CI)	*p*	OR (95%CI)	*p*	OR (95%CI)	*p*
TyG	1.26 (1.19–1.33)	<0.001	1.26 (1.20–1.33)	<0.001	1.23 (1.16–1.30)	<0.001
TyG-BMI	1.13 (1.07–1.19)	<0.001	1.17 (1.10–1.23)	<0.001	1.11 (1.05–1.18)	<0.001
TyG-WC	1.25 (1.18–1.32)	<0.001	1.25 (1.18–1.32)	<0.001	1.20 (1.13–1.27)	<0.001
TyG-WhtR	1.05 (1.01–1.11)	0.066	1.13 (1.07–1.20)	<0.001	1.07 (1.01–1.14)	0.025
TyG						
Q1	1 (Reference)		1 (Reference)		1 (Reference)	
Q2	1.09 (0.92–1.28)	0.319	1.06 (0.90–1.25)	0.492	1.10 (0.93–1.30)	0.278
Q3	0.10 (0.81–1.13)	0.585	0.90 (0.77–1.07)	0.235	0.89 (0.75–1.06)	0.177
Q4	1.55 (1.33–1.81)	<0.001	1.51 (1.29–1.76)	<0.001	1.45 (1.23–1.71)	<0.001
P for trend	<0.001		<0.001		<0.001	
TyG-BMI						
Q1	1 (Reference)		1 (Reference)		1 (Reference)	
Q2	0.90 (0.76–1.05)	0.179	0.95 (0.81–1.12)	0.520	0.96 (0.81–1.14)	0.644
Q3	0.96 (0.82–1.12)	0.603	1.02 (0.87–1.20)	0.796	1.04 (0.88–1.23)	0.666
Q4	1.27 (1.09–1.48)	0.002	1.38 (1.18–1.62)	<0.001	1.33 (1.12–1.58)	0.001
P for trend	<0.001		<0.001		<0.001	
TyG-WC						
Q1	1 (Reference)		1 (Reference)		1 (Reference)	
Q2	1.10 (0.93–1.30)	0.254	1.11 (0.94–1.31)	0.221	1.15 (0.97–1.37)	0.106
Q3	1.15 (0.98–1.36)	0.087	1.14 (0.97–1.35)	0.115	1.18 (0.99–1.40)	0.058
Q4	1.70 (1.45–1.98)	<0.001	1.67 (1.43–1.95)	<0.001	1.64 (1.38–1.95)	<0.001
P for trend	<0.001		<0.001		<0.001	
TyG-WhtR						
Q1	1 (Reference)		1 (Reference)		1 (Reference)	
Q2	0.85 (0.73–0.99)	0.039	0.94 (0.80–1.10)	0.453	0.95 (0.81–1.12)	0.540
Q3	0.79 (0.67–0.93)	0.003	0.90 (0.77–1.06)	0.195	0.91 (0.76–1.07)	0.245
Q4	1.07 (0.92–1.24)	0.378	1.28 (1.09–1.49)	0.002	1.25 (1.05–1.48)	0.012
P for trend	0.290		0.001		<0.004	

OR, odd ratio; CI, confidence interval; BMI, body mass index; TyG, triglyceride-glucose index; WC: waist circumference; WHtR: waist-to-height ratio; Q: quartile. Model 1: Unadjusted. Model 2: age and gender were adjusted; model 3: age, gender, residence, marriage, education level, smoking, drinking, hypertension, dyslipidemia and diabetes were adjusted.

In an analysis stratified by quartiles of the TyG index and its related indices—TyG-BMI, TyG-WC, and TyG-WHtR—individuals in the highest quartile (Q4) exhibited significantly elevated ORs for ADL Limited compared to those in the lowest quartile (Q1). The adjusted ORs (95% CI) for Q4 were as follows: 1.45(1.23–1.71) for TyG, 1.33 (1.12–1.58) for TyG-BMI, 1.64 (1.38–1.95) for TyG-WC, and 1.25 (1.05–1.48) for TyG-WHtR, respectively, and the trend test confirmed this association as statistically significant (*p* < 0.001).

### 3.3 Associations of TyG and its related parameters with ADL-IADL limited

Table 3 illustrates the statistical associations between the TyG index and its associated parameters—TyG-BMI, TyG-WC, and TyG-WHtR—with ADL-IADL Limited. Logistic regression analysis, adjusted for age and sex in model 2, revealed a positive correlation between elevated indices of TyG and its related parameters and the likelihood of ADL-IADL Limited. These significant associations were sustained after additional adjustments for a comprehensive set of covariates, including residence, marital status, education level, smoking, alcohol consumption, hypertension, dyslipidemia, and diabetes, as per model 3. The adjusted odds ratios (ORs) for the TyG index in relation to ADL-IADL Limited were 1.20 (95% CI: 1.14–1.26, *p* < 0.001). For the TyG-BMI index, the adjusted OR was 1.13 (95% CI:1.08–1.19, *p* < 0.001), also indicating a significant association, as did the TyG-WC index with an adjusted OR of 1.14 (95% CI:1.09–1.20, *p* < 0.001). The TyG-WHtR index showed a slightly higher adjusted OR of 1.13 (95% CI:1.07 to 1.18, *p* < 0.001), which was likewise statistically significant.

**Table 3:** Associations of TyG and its related parameters with ADL-IADL limited.

Characteristics	Model 1		Model 2		Model 3	
	OR (95%CI)	*p*	OR (95%CI)	*p*	OR (95%CI)	*p*
TyG	1.20 (1.15–1.25)	<0.001	1.20 (1.15–1.25)	<0.001	1.20 (1.14–1.26)	<0.001
TyG-BMI	1.13 (1.08–1.18)	<0.001	1.13 (1.08–1.18)	<0.001	1.13 (1.08–1.19)	<0.001
TyG-WC	1.14 (1.09–1.19)	<0.001	1.14 (1.09–1.19)	<0.001	1.14 (1.09–1.20)	<0.001
TyG-WhtR	1.11 (1.07–1.16)	<0.001	1.12 (1.07–1.17)	<0.001	1.13 (1.07–1.18)	<0.001
TyG						
Q1	1 (Reference)		1 (Reference)		1 (Reference)	
Q2	1.10 (0.96–1.25)	0.164	1.09 (0.96–1.25)	0.187	1.11 (0.97–1.27)	0.131
Q3	1.17 (1.03–1.33)	0.018	1.16 (1.02–1.32)	0.026	1.19 (1.04–1.35)	0.014
Q4	1.46 (1.29–1.66)	<0.001	1.45 (1.28–1.65)	<0.001	1.46 (1.28–1.67)	<0.001
P for trend	<0.001		<0.001		<0.001	
TyG-BMI						
Q1	1 (Reference)		1 (Reference)		1 (Reference)	
Q2	0.97 (0.85–1.11)	0.671	0.97 (0.85–1.11)	0.686	0.97 (0.85–1.11)	0.662
Q3	1.12 (0.99–1.28)	0.080	1.12 (0.98–1.28)	0.086	1.11 (0.97–1.27)	0.117
Q4	1.29 (1.13–1.46)	<0.001	1.28 (1.13–1.46)	<0.001	1.28 (1.12–1.48)	<0.001
P for trend	<0.001		<0.001		<0.001	
TyG-WC						
Q1	1 (Reference)		1 (Reference)		1 (Reference)	
Q2	1.09 (0.96–1.24)	0.193	1.09 (0.96–1.24)	0.206	1.08 (0.95–1.23)	0.257
Q3	1.06 (0.93–1.20)	0.419	1.05 (0.92–1.20)	0.477	1.05 (0.91–1.20)	0.520
Q4	1.37 (1.20–1.55)	<0.001	1.36 (1.19–1.54)	<0.001	1.35 (1.18–1.54)	<0.001
P for trend	<0.001		<0.001		<0.001	
TyG-WhtR						
Q1	1 (Reference)		1 (Reference)		1 (Reference)	
Q2	0.98 (0.86–1.11)	0.721	0.99 (0.87–1.12)	0.838	0.98 (0.86–1.12)	0.784
Q3	1.01 (0.89–1.15)	0.862	1.02 (0.90–1.17)	0.732	1.03 (0.90–1.18)	0.652
Q4	1.31 (1.15–1.49)	<0.001	1.33 (1.17–1.51)	<0.001	1.33 (1.16–1.53)	<0.001
P for trend	<0.001		<0.001		<0.001	

OR, odd ratio; CI, confidence interval; BMI, body mass index; TyG, triglyceride-glucose index; WC: waist circumference; WHtR: waist-to-height ratio; Q: quartile. Model 1: Unadjusted. Model 2: age and gender were adjusted; model 3: age, gender, residence, marriage, education level, smoking, drinking, hypertension, dyslipidemia and diabetes were adjusted.

Table 3 delineates the significant associations between the highest (Q4) and lowest (Q1) quartiles of the TyG index and its related parameters—TyG-BMI, TyG-WC, and TyG-WHtR—with ADL-IADL Limited. Individuals in Q4 exhibited adjusted odds ratios (ORs) (95% CI) of 1.46 (1.28–1.67) for TyG, 1.28 (1.12–1.48) for TyG-BMI, 1.35 (1.18–1.54) for TyG-WC, and 1.33 (1.16–1.53) for TyG-WHtR, respectively, when compared to those in Q1, and the trend test confirmed this association as statistically significant (*p* < 0.001).

### 3.4 Associations between TyG index and its related parameters with the risk of ADL and ADL-IADL were evaluated by RCS

**Figure 2:**
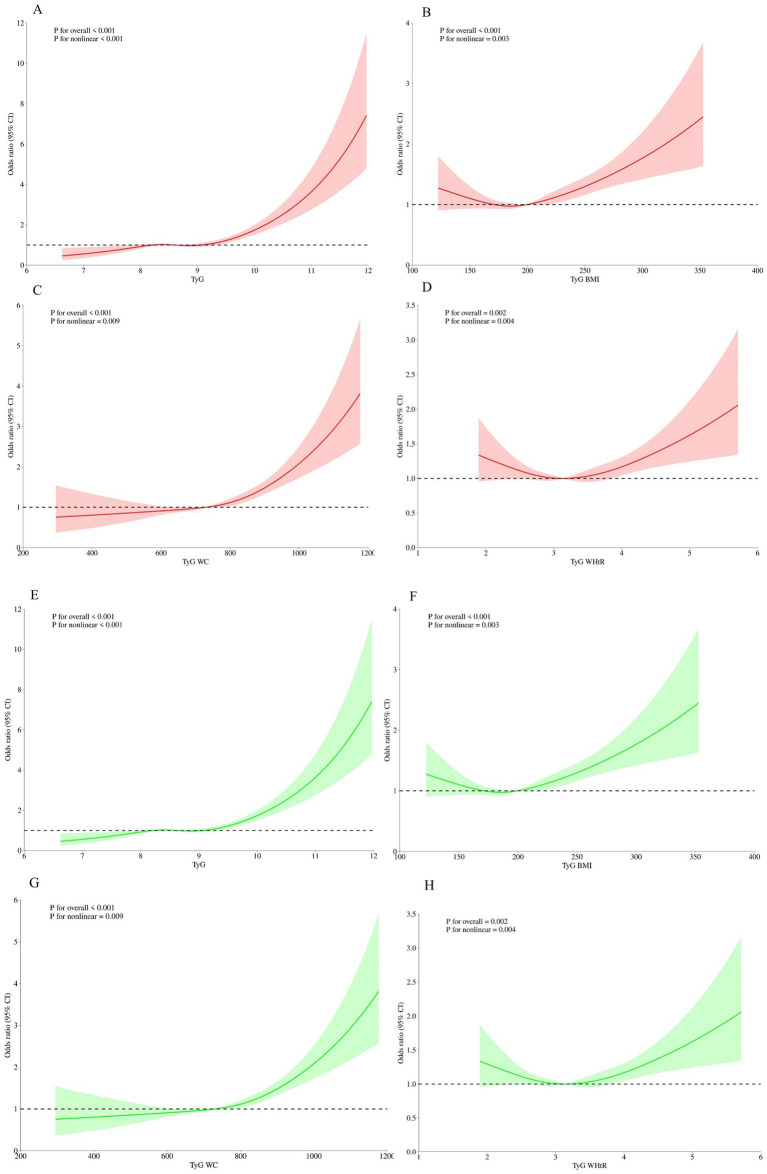
Associations between TyG index and its related parameters with the risk of ADL limited or ADL-IADL limited were evaluated by RCS. The solid red lines correspond to the central estimates, and the red-shaded regions indicate the 95% confidence intervals in ADL limited. The solid green lines correspond to the central estimates, and the green-shaded regions indicate the 95% confidence intervals in ADL-IADL limited. Age, gender, residence, marriage, education level, smoking, drinking, hypertension, dyslipidemia and diabetes were adjusted. **(A)** TyG index in ADL; **(B)** TyG-BMI index in ADL; **(C)** TyG-WC index in ADL; **(D)** TyG-WHtR index in ADL; **(E)** TyG index in ADL-IADL; **(F)** TyG-BMI index in ADL-IADL;**(G)** TyG-WC index in ADL -IADL; **(H)** TyG-WHtR index in ADL-IADL. BMI, body mass index; CI, confidence intervals; RCS, restricted cubic spline; TyG, triglyceride-glucose; WC, waist circumference; WhtR, waist-to-height ratio.

We conducted restricted cubic splines (RCS) to flexibly model and visualize the associations of TyG-related indices with ADL and ADL-IADL Limited patients (Figure 2). After adjusting for all covariates in model 3 described above, we observed non-linear trends in the relationships between TyG, TyG-BMI, TyG-WC, and TyG-WHtR indices with ADL Limited (non-linear P: 0.001, 0.003, 0.009, and 0.004, respectively) (Figures 2A–D). Similarly, a non-linear association was observed between TyG and ADL-IADL Limited (non-linear *p* = 0.011). Linear trends were observed in the relationships between TyG-BMI, TyG-WC, and TyG-WHtR indices with ADL-IADL Limited (non-linear P: 0.415, 0.083, and 0.464, respectively) (Figures 2E–H).

### 3.5 Subgroup analyses of association between TyG index and its related parameters among ADL limited

**Figure 3:**
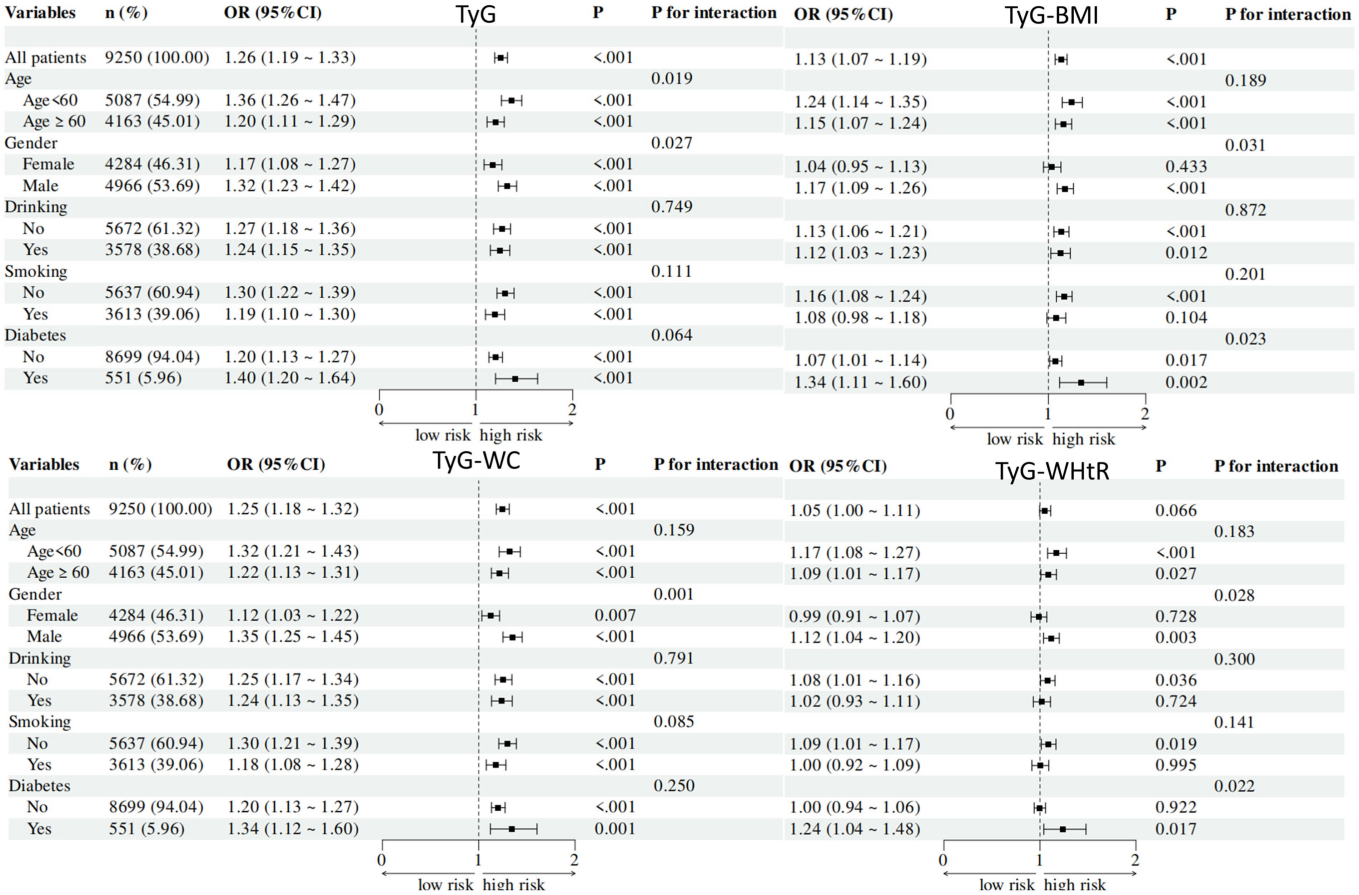
Subgroup analyses of association between TyG index and its related parameters among ADL limited. Subgroup analyses of association between TyG-related indices and among ADL limited. Black box means OR value, and the bars on both sides of box mean 95% CI of OR. CI, confidence intervals; OR, odd ratios; BMI, body mass index; TyG, triglyceride-glucose; WC, waist circumference; WhtR, waist-to-height ratio.

To delve deeper into the relationship between TyG-related indices and the presence of ADL Limited, we conducted a stratified analysis of the ADL population based on age, sex, smoking status, alcohol consumption, and diabetes history (Figure 3). The stratified analysis revealed significant associations between TyG, TyG-BMI, TyG-WC, and TyG-WHtR indices and the likelihood of ADL Limited. Notably, in the female, smoker subgroup, the effect of TyG-BMI on ADL restriction was not statistically significant. The effect of TyG-WHtR on ADL restriction was not statistically significant in the female, smokers, drinkers, and no diabetes subgroup.

The interaction analysis revealed significant differences in several variables across different risk groups for the TyG index. Specifically, age demonstrated a significant interaction effect (P for interaction = 0.019), gender also showed a significant interaction effect (P for interaction = 0.027). For the TyG-BMI index, gender was the only variable that showed a significant interaction effect (P for interaction = 0.031). The TyG-WC index showed significant interaction effects for gender (P for interaction = 0.001). The TyG-WHtR index showed significant interaction effects for gender (P for interaction = 0.028) and diabetes status (P for interaction = 0.022).

### 3.6 Subgroup analyses of association between TyG-related indices among ADL-IADL

**Figure 4:**
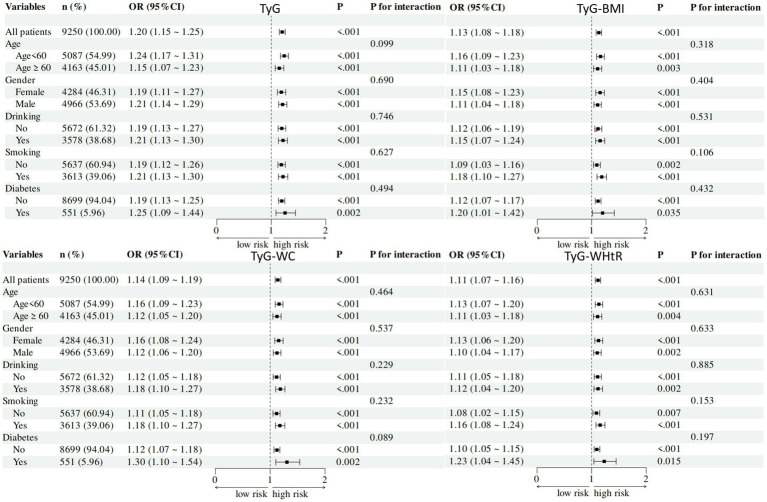
Subgroup analyses of association between TyG-related indices among ADL-IADL limited. Subgroup analyses of association between TyG-related indices and among ADL-IADL limited. Black box means OR value, and the bars on both sides of box mean 95% CI of OR. CI, confidence intervals; OR, odd ratios; BMI, body mass index; TyG, triglyceride-glucose; WC, waist circumference; WhtR, waist-to-height ratio.

To delve deeper into the relationship between TyG-related indices and the presence of ADL-IADL Limited, we conducted a stratified analysis of the ADL-IADL population based on age, sex, smoking status, alcohol consumption, and diabetes history (Figure 4). The stratified analysis revealed significant associations between TyG, TyG-BMI, TyG-WC, and TyG-WHtR indices and the likelihood of ADL-IADL Limited.

### 3.7 Sensitivity analysis

In a sensitivity analysis designed to assess the robustness of our findings, the exclusion of participants over the age of 80 did not significantly modify the positive association observed between TyG-related indices and the risk of ADL Limited and ADL-IADL Limited (Supplementary Tables S2, S3). This sensitivity analysis confirms the stability of the associations between the TyG index and its related parameters with the risk of functional limitations, suggesting that the observed relationships are not substantially influenced by the presence of the oldest old in the study population. Propensity score matching (PSM) also yielded comparable outcomes (Supplementary Tables S5, S6). Adjusting for physical activity and inflammatory markers on the basis of Model 3 did not significantly alter the positive association observed between TyG-related indices and the risk of ADL and ADL-IADL limitations (Supplementary Tables S7, S8). Excluding individuals with a BMI > 40 did not significantly alter the positive association observed between TyG-related indices and the risk of ADL and ADL-IADL limitations (Supplementary Tables S9, S10).

## 4 Discussion

Our investigation, leveraging a comprehensive national cohort, presents an initial exploration of the relationships between various TyG indices and risks related to ADL Limited or ADL-IADL Limited. We found that elevated levels of TyG-related indices were independently linked to an increased risk of limitations in ADL and ADL-IADL. The RCS curve analysis revealed a non-linear positive association between TyG-related indices and the risk of ADL limited, while a linear positive association was observed for the risk of ADL-IADL limited. Additionally, subgroup analyses indicated a more pronounced association between the TyG-WC index and ADL limited, and between the TyG index and ADL-IADL limited. In conclusion, our study suggests that TyG-related indices could potentially serve as significant surrogate biomarkers for the clinical monitoring and management of individuals with limitations in ADL and ADL-IADL.

In the general population, individuals with limitations in ADL or ADL-IADL represent a significant demographic, imposing considerable societal and healthcare burdens. Aligning with previous findings, our study reports a 16.3% prevalence of ADL Limited and a notably higher rate of 28.3% for ADL-IADL Limited (Millán-Calenti et al., 2010). Identifying modifiable prognostic factors and risk elements is crucial for refining management strategies and improving outcomes for these individuals. Individuals with ADL and ADL-IADL limitations frequently present with insulin resistance, glucose and lipid metabolism dysregulation, and increased inflammatory responses (Pei et al., 2022). The TyG index has been recognized as an accessible and dependable indicator for the detection of insulin resistance and the assessment of cardiovascular risk in the general population (Lopez-Jaramillo et al., 2023). Although research applying these biomarkers to screen for ADL and ADL-IADL limitations is relatively sparse, our study offers novel insights into potential risk factors that could be pivotal for early identification and intervention in this vulnerable population.

In our investigation, elevated TyG index values were found to be associated with an increased risk of both ADL Limited and ADL-IADL Limited, persisting even after accounting for potential confounding factors. Although the direct relationship between the TyG index and the risk of ADL and ADL-IADL limitations has not been previously established in the literature, there is substantial evidence indicating that a high TyG index is associated with reduced muscle mass or sarcopenia (Ahn et al., 2020; Chen et al., 2022; Kim et al., 2021; Zheng et al., 2022). The decline in muscle mass and strength is recognized as a significant factor contributing to functional impairment in the elderly population (Vermeulen et al., 2011; Jonkman et al., 2018), Muscle mass loss is a well-documented precursor to the development of limitations in both ADL and IADL (Wang et al., 2020). Elderly individuals with diminished muscle mass often encounter greater difficulty in executing tasks essential for ADL and IADL (Visser et al., 2005; Meskers et al., 2019). A previous cross-sectional study has demonstrated a correlation between muscle mass—defined by muscle strength or power relative to muscle mass—and a decline in ADL performance (Barbat-Artigas et al., 2012). Ahn et al. have reported a positive association between an elevated TyG index and an increased risk of low skeletal muscle mass index in a Korean adult population. This finding is corroborated by other studies, which have identified high TyG index levels as a risk factor for reduced muscle mass or strength (Chen et al., 2022; Kim et al., 2021), with implications extending to younger demographics, including adolescents between the ages of 12 to 18 years (Zhou et al., 2022). Consequently, the extant literature indirectly substantiates the link between the TyG index and the presence of ADL limitations, aligning with the outcomes of our study. Nonetheless, the majority of these studies have evaluated the TyG index at a single time point, which may not accurately capture the chronic exposure typically associated with the development of ADL limitations. In light of the findings from the current study, there is a pronounced requirement for additional prospective, longitudinal research endeavors that can delineate the long-term dynamics between TyG index trajectories and changes in physical function. Such studies will provide a more definitive understanding of the temporal associations and potentially reveal novel avenues for intervention and prevention strategies in elderly care.

To date, the precise biological mechanisms that mediate the relationship between the TyG index and limitations in ADL and ADL-IADL have not been fully elucidated. During the aging process, the decline in muscle mass is known to result in diminished peripheral glucose uptake, which in turn can precipitate a state of hyperinsulinemia and insulin resistance (Consitt and Clark, 2018). Furthermore, the physical inactivity often associated with older age is known to exacerbate insulin resistance (Amati et al., 2009). The aging process also encompasses a range of alterations, including chronic inflammation, impaired glycogen synthesis, and disruptions in oxidative pathways, all of which can contribute to the dysregulation of insulin homeostasis (Soysal et al., 2016). Insulin resistance has been shown to negatively impact muscle protein turnover, leading to reduced muscle mass and the development of sarcopenia, which is a significant contributor to functional impairment (Siew et al., 2007; Fried et al., 2001). Consequently, the interplay between insulin resistance and diminished muscle mass disrupts energy metabolism and physical capacity, consequently elevating the risk of functional decline.

In our study, elevated values of the TyG index were associated with a heightened risk of experiencing limitations in ADL and ADL-IADL, persisting after adjusting for confounding factors. This finding is in concordance with a survey conducted among Polish Caucasians, which identified significant associations between abdominal obesity, elevated triglyceride levels, and increased disability rates (Puzianowska-Kuznicka et al., 2022). Additionally, research among American post-cardiac surgery patients aged 50 to 79 demonstrated a link between BMI of 30 kg/m^2^ or higher and greater odds of ADL decline (Gaulton and Neuman, 2018). The risk of ADL limitations was found to escalate progressively with overweight and obesity status compared to individuals of normal weight (Backholer et al., 2012; Reynolds and McIlvane, 2009; Armour et al., 2013; Vincent et al., 2010).

Several potential mechanisms may underlie the observed association between obesity measures and functional limitations in ADL. Initially, increments in body mass index (BMI) or waist circumference (WC) could precipitate severe chronic conditions that culminate in disability. Secondly, existing literature supports a link between obesity and a decline in physical function (Vincent et al., 2010); specifically, abdominal obesity may negatively impact joint mechanics, heightening the risk of falls (Corbeil et al., 2001) and impairing the ability to perform daily activities. Thirdly, obesity is known to be associated with low-grade systemic inflammation and oxidative stress (Ferrucci et al., 2002), which can erode muscle mass and grip strength (Howard et al., 2007), thus contributing to a decrement in physical function (Brinkley et al., 2009). Moreover, elevated waist-to-height ratio (WHtR) levels have been associated with adverse effects on ADL limitations (Ramírez-Vélez et al., 2020).

A study has indicated that patients with type 2 diabetes mellitus, particularly those with comorbid atrial fibrillation (AF), exhibit a higher prevalence of cognitive impairment, which is directly associated with a decline in ADL (Militaru et al., 2024). This decline may be multifactorial, encompassing not only the direct impact of type 2 diabetes on metabolic regulation but also indirect effects through increased inflammation, oxidative stress, and microvascular complications, which can lead to muscle weakness and reduced physical performance. A study has indicated that insulin resistance can accelerate muscle protein breakdown: the activation of the ubiquitin-proteasome pathway through defects in muscle cell signaling, which may lead to a decrease in muscle mass and strength (Kim, 2025). Studies have indicated that mitochondrial dysfunction can lead to an increase in reactive oxygen species (ROS) and may disrupt carbohydrate and lipid metabolism, events associated with cancer-induced muscle loss (Gicquel et al., 2024). A study has indicated that sarcopenia is directly associated with limitations in ADL limited (Gao et al., 2021).

This study, targeting an elderly Chinese demographic, it examines the influence of the TyG index and its combined obesity indices on the prevalence of limitations in ADL and ADL-IADL. Our research stands as the pioneering inquiry into the aggregate effects of the TyG index and its combined obesity indices in patients presenting with ADL Limited and ADL-IADL Limited conditions. The findings underscore a significant association, indicating that the confluence of TyG with obesity indices is linked to an amplified risk of ADL Limited and ADL-IADL Limited. RCS analysis, the study discerns that the relationships predominantly follow non-linear trajectories. However, within the context of ADL-IADL Limited, linear correlations are observed with TyG-BMI, TyG-WC, and TyG-WHtR indices. It is posited that these results may be shaped by a constellation of factors, including the size of the sample analyzed, the clinical complexity of the conditions in question, the breadth of data dimensions, the configuration of RCS curve parameters, and the choice of statistical models.

Our study leveraged a large-scale, prospective cohort from the Chinese population to examine the relationship between TyG-related indices and the prevalence of limitations in ADL or combined ADL and ADL-IADL. It is important to highlight that recent studies have posed a critical question concerning the most suitable cutoff values for TyG-related indices within different populations. Consequently, there is an imperative need for further research to identify the optimal cutoff points for these emerging biomarkers in relation to ADL Limited and ADL-IADL Limited patients, taking into account the variability across diverse ethnic groups. Identifying such cutoff points will enhance the clinical utility of TyG-related indices as predictive tools in geriatric care and potentially across other demographic groups. The causal relationship between TyG-related indices and dysfunction could be explored in the future through longitudinal cohort studies or using Mendelian randomisation methods.

Specifically, elevated TyG indices, particularly the TyG-WC index for ADL Limited and the TyG index for ADL-IADL Limited, could be used as early indicators to guide targeted interventions aimed at improving insulin sensitivity and reducing the risk of functional decline. In terms of patient care, our findings highlight the importance of addressing insulin resistance and metabolic health in elderly individuals with functional limitations. Interventions such as lifestyle modifications, including diet and exercise, could be prioritized for individuals with elevated TyG indices to potentially mitigate the risk of further functional decline.

This study has the following strengths and limitations. Firstly, it evaluated the association between TyG-related indices and increased risk of ADL Limited and ADL-IADL Limited patients. These findings provide evidence that TyG-related indices may serve as risk factors for ADL Limited and ADL-IADL Limited patients. Secondly, the study design based on a large-scale national population provides robust evidence linking TyG-related indices with ADL Limited and ADL-IADL Limited patients. Additionally, we controlled for a range of covariates to determine the independent associations between TyG-related indices and ADL Limited and ADL-IADL Limited patients.

However, several limitations should be noted in this study. Firstly, anthropometric measurements and blood indices were collected only at baseline. Secondly, being an observational study, inherent biases such as residual confounding factors such as dietary that were not fully measured cannot be entirely ruled out. Therefore, further research is needed to validate our findings. Lastly, the study included patients exclusively from China, thus the actual clinical applicability of TyG-related indices in other countries and different ethnicities requires further validation.

## 5 Conclusion

In conclusion, there is a significant association between TyG-related indices and increased risk of ADL Limited and ADL-IADL Limited. Our study findings suggest that TyG-related indices can assist clinicians in making better clinical decisions for ADL Limited and ADL-IADL Limited patients during long-term follow-up.

## Data Availability

The raw data supporting the conclusions of this article will be made available by the authors, without undue reservation.
