# Supplementary material for: Association of triglyceride-glucose index and its related parameters with functional disability: evidence from the China Health and Retirement Longitudinal Study
Source: Front Aging Neurosci. 2025 May 19;17:1488124. doi: 10.3389/fnagi.2025.1488124 (PMC12127429; doi:10.3389/fnagi.2025.1488124)
Supplement: Supplementary file 1 [file Table_1.docx]

**Association of triglyceride-glucose index and its related parameters with functional disability: evidence from the China Health and Retirement Longitudinal Study (CHARLS)**

**Supplementary Materials**

Table S1 Baseline characteristics of participants with ADL and ADL-IADL, excluding individuals over 80 years of age .

| Variables | ADL Unlimited  (n = 7600) | ADL Limited  (n = 1446) | *P* | ADL-IADL Unlimited  (n = 6478) | ADL-IADL Limited  (n = 2568) | *P* |
| --- | --- | --- | --- | --- | --- | --- |
| Age, Mean ± SD | 57.92 ± 8.95 | 61.98 ± 9.05 | <.001 | 58.50 ± 9.08 | 58.74 ± 9.13 | 0.245 |
| Gender, Male(%) | 4011 (52.78) | 852 (58.92) | <.001 | 3450 (53.26) | 1413 (55.02) | 0.129 |
| Marriage, n(%) | 6785 (89.28) | 1230 (85.06) | <.001 | 5748 (88.73) | 2267 (88.28) | 0.542 |
| Rural, n(%) | 4867 (64.04) | 1043 (72.13) | <.001 | 4246 (65.54) | 1664 (64.80) | 0.501 |
| Drinking, n(%) | 2965 (39.01) | 538 (37.21) | 0.196 | 2489 (38.42) | 1014 (39.49) | 0.349 |
| Smoking, n(%) | 2977 (39.17) | 544 (37.62) | 0.268 | 2561 (39.53) | 960 (37.38) | 0.059 |
| Educational, n(%) |  |  | <.001 |  |  | 0.400 |
| Primary and below | 3411 (44.92) | 894 (61.83) |  | 3056 (47.20) | 1249 (48.67) |  |
| Junior secondary school | 1728 (22.75) | 291 (20.12) |  | 1475 (22.78) | 544 (21.20) |  |
| High school and above | 2455 (32.33) | 261 (18.05) |  | 1943(30.02) | 773 (30.12) |  |
| TyG, M (Q₁, Q₃) | 8.59 (8.22, 9.02) | 8.67 (8.28, 9.22) | <.001 | 8.58 (8.21, 9.02) | 8.66 (8.28, 9.14) | <.001 |
| TyG BMI, M (Q₁, Q₃) | 199.13 (175.76, 227.47) | 204.08 (176.74, 236.97) | <.001 | 198.50 (175.53, 226.88) | 203.55 (177.31, 233.60) | <.001 |
| TyG WC, M (Q₁, Q₃) | 725.68 (652.66, 811.33) | 750.04 (666.57, 849.93) | <.001 | 726.84 (652.94, 811.30) | 737.46 (660.89, 831.18) | <.001 |
| TyG WHtR, M (Q₁, Q₃) | 3.15 (2.78, 3.60) | 3.16 (2.75, 3.73) | 0.154 | 3.14 (2.77, 3.59) | 3.21 (2.79, 3.70) | <.001 |
| Hypertension, n(%) | 1849 (24.33) | 492 (34.02) | <.001 | 1670 (25.78) | 671 (26.13) | 0.732 |
| Diabetes, n(%) | 400 (5.26) | 139 (9.61) | <.001 | 361 (5.57) | 178 (6.93) | 0.014 |
| Dyslipidemia, n(%) | 688 (9.19) | 180 (12.67) | <.001 | 613 (9.61) | 255 (10.10) | 0.488 |

Abbreviation: ADL, Activity of Daily Living. IADL, Instrumental Activity of Daily Living . TyG, Triglyceride and glucose.BMI, Body Mass Index.WC, Waist Circumference. WhtR, Waist-to-Height Ratio.

Table S2 Associations of TyG and its related parameters with ADL, excluding individuals over 80 years of age.

| Variables | Model1 | |  | Model2 | |  | Model3 | |
| --- | --- | --- | --- | --- | --- | --- | --- | --- |
|  | OR (95%CI) | *P* |  | OR (95%CI) | *P* |  | OR (95%CI) | *P* |
| TyG | 1.42 (1.31 - 1.53) | <0.001 |  | 1.42 (1.31 - 1.54) | <0.001 |  | 1.39 (1.27 - 1.51) | <0.001 |
| TyG-BMI | 1.03 (1.02 - 1.05) | <0.001 |  | 1.04 (1.03 - 1.06) | <0.001 |  | 1.04 (1.02 - 1.05) | <0.001 |
| TyG -WC | 1.02 (1.01 - 1.02) | <0.001 |  | 1.02 (1.01 - 1.02) | <0.001 |  | 1.02 (1.01 - 1.02) | <0.001 |
| TyG -WhtR | 1.11 (1.01 - 1.21) | 0.025 |  | 1.23 (1.12 - 1.34) | <0.001 |  | 1.22 (1.10- 1.34) | <0.001 |
| TyG |  |  |  |  |  |  |  |  |
| Q1 | 1.000 (Reference) |  |  | 1.000 (Reference) |  |  | 1.000 (Reference) |  |
| Q2 | 1.07 (0.91 - 1.26) | 0.42 |  | 1.04 (0.88 - 1.23) | 0.662 |  | 1.06 (0.89 - 1.25) | 0.511 |
| Q3 | 0.95 (0.80 - 1.12) | 0.53 |  | 0.90 (0.76 - 1.06) | 0.202 |  | 0.87(0.73 - 1.04) | 0.118 |
| Q4 | 1.55 (1.33 - 1.81) | <0.001 |  | 1.50 (1.28- 1.76) | <0.001 |  | 1.44 (1.22 - 1.70) | <.001 |
| P for trend | <0.001 |  |  | <0.001 |  |  | <0.001 |  |
| TyG -BMI |  |  |  |  |  |  |  |  |
| Q1 | 1.000 (Reference) |  |  | 1.000 (Reference) |  |  | 1.000 (Reference) |  |
| Q2 | 0.88 (0.75 - 1.04) | 0.123 |  | 0.92 (0.78 - 1.09) | 0.344 |  | 0.94 (0.79 - 1.11) | 0.456 |
| Q3 | 0.96 (0.81 - 1.13) | 0.596 |  | 1.04 (0.85 - 1.18) | 0.963 |  | 1.03 (0.86 - 1.22) | 0.770 |
| Q4 | 1.30 (1.11 - 1.51) | 0.001 |  | 1.38 (1.18 - 1.62) | <.001 |  | 1.35 (1.14 - 1.61) | <0.001 |
| P for trend | <0.001 |  |  | <0.001 |  |  | <0.001 |  |
| TyG -WC |  |  |  |  |  |  |  |  |
| Q1 | 1.000 (Reference) |  |  | 1.000 (Reference) |  |  | 1.000 (Reference) |  |
| Q2 | 1.10 (0.93 - 1.30) | 0.268 |  | 1.11 (0.93 - 1.31) | 0.243 |  | 1.14 (0.96 - 1.35) | 0.144 |
| Q3 | 1.14 (0.97 - 1.35) | 0.115 |  | 1.13 (0.95 - 1.34) | 0.162 |  | 1.17 (0.98- 1.39) | 0.086 |
| Q4 | 1.71 (1.47 - 2.01) | <0.001 |  | 1.67 (1.43 - 1.96) | <0.001 |  | 1.66 (1.40 - 1.97) | <0.001 |
| P for trend | <0.001 |  |  | <0.001 |  |  | <0.001 |  |
| TyG -WhtR |  |  |  |  |  |  |  |  |
| Q1 | 1.000 (Reference) |  |  | 1.000 (Reference) |  |  | 1.000 (Reference) |  |
| Q2 | 0.88 (0.75 - 1.03) | 0.105 |  | 0.96 (0.82 - 1.13) | 0.641 |  | 0.98 (0.83 - 1.16) | 0.842 |
| Q3 | 1.31 (1.08 - 1.94) | 0.007 |  | 0.90 (0.77 - 1.07) | 0.232 |  | 0.92 (0.77 - 1.09) | 0.309 |
| Q4 | 1.13 (0.97 - 1.31) | 0.128 |  | 1.32 (1.13 - 1.55) | <.001 |  | 1.31 (1.11 - 1.56) | 0.002 |
| P for trend | 0.100 |  |  | <0.001 |  |  | <0.001 |  |
| Abbreviation: OR, odd ratio; CI, confidence interval; BMI, body mass index; TyG, triglyceride-glucose index; WC: waist circumference; WHtR: waist-toheight ratio . Q: quartile . Model 1: unadjusted .Model 2: age and gender were adjusted; model 3: age, gender, rural, marriage, education level, smoking , drinking, hypertension, Dyslipidemia and diabetes were adjusted. | | | | | | | | |

Table S3 Associations of TyG and its related parameters with ADL-IADL, excluding individuals over 80 years of age.

| Variables | Model1 | |  | Model2 | |  | Model3 | |
| --- | --- | --- | --- | --- | --- | --- | --- | --- |
|  | OR (95%CI) | *P* |  | OR (95%CI) | *P* |  | OR (95%CI) | *P* |
| TyG | 1.30 (1.21 - 1.39) | <0.001 |  | 1.29 (1.21 - 1.38) | <00.001 |  | 1.30(1.21 - 1.39) | <0.001 |
| TyG -BMI | 1.03 (1.02 - 1.04) | <0.001 |  | 1.03 (1.02 - 1.04) | <0.001 |  | 1.03 (1.02 - 1.05) | <0.001 |
| TyG - WC | 1.02 (1.01 - 1.03) | <0.001 |  | 1.02 (1.01 - 1.03) | <0.001 |  | 1.02 (1.01 - 1.03) | <0.001 |
| TyG- WhtR | 1.11 (1.02 - 1.27) | <0.001 |  | 1.20 (1.12 - 1.29) | <0.001 |  | 1.20 (1.11 - 1.30) | <0.001 |
| TyG |  |  |  |  |  |  |  |  |
| Q1 | 1.000 (Reference) |  |  | 1.000 (Reference) |  |  | 1.000 (Reference) |  |
| Q2 | 1.07 (0.94 - 1.22) | 0.306 |  | 1.07 (0.93 - 1.22) | 0.338 |  | 1.08 (0.94 - 1.23) | 0.26 |
| Q3 | 1.16 (1.01 - 1.32) | 0.031 |  | 1.15 (1.05 - 1.31) | 0.042 |  | 1.16 (1.02 - 1.33) | 0.026 |
| Q4 | 1.43 (1.26 - 1.63) | <0.001 |  | 1.42 (1.25 - 1.62) | <0.001 |  | 1.42 (1.24 - 1.63) | <0.001 |
| P for trend | <0.001 |  |  | <0.001 |  |  | <0.001 |  |
| TyG -BMI |  |  |  |  |  |  |  |  |
| Q1 | 1.000 (Reference) |  |  | 1.000 (Reference) |  |  | 1.000 (Reference) |  |
| Q2 | 0.93 (0.81 - 1.06) | 0.26 |  | 0.93 (0.81 - 1.06) | 0.271 |  | 0.93 (0.81 - 1.06) | 0.295 |
| Q3 | 1.10 (0.97 - 1.25) | 0.152 |  | 1.10 (0.97 - 1.25) | 0.155 |  | 1.09(0.95 - 1.25) | 0.211 |
| Q4 | 1.26(1.11 - 1.43) | <0.001 |  | 1.26 (1.10 - 1.43) | <0.001 |  | 1.25 (1.01 - 1.44) | 0.002 |
| P for trend | <0.001 |  |  | <0.001 |  |  | <0.001 |  |
| TyG -WC |  |  |  |  |  |  |  |  |
| Q1 | 1.000 (Reference) |  |  | 1.000 (Reference) |  |  | 1.000 (Reference) |  |
| Q2 | 1.08 (0.95- 1.23) | 0.258 |  | 1.08(0.94 - 1.23) | 0.272 |  | 1.08 (0.95 - 1.23) | 0.259 |
| Q3 | 1.04 (0.91 - 1.19) | 0.545 |  | 1.04(0.91 - 1.18) | 0.598 |  | 1.04 (0.91 - 1.19) | 0.590 |
| Q4 | 1.36 (1.20 - 1.55) | <0.001 |  | 1.35 (1.19 - 1.54) | <.001 |  | 1.34 (1.17 - 1.54) | <.001 |
| P for trend | <0.001 |  |  | <0.001 |  |  | <0.001 |  |
| TyG -WhtR |  |  |  |  |  |  |  |  |
| Q1 | 1.000 (Reference) |  |  | 1.000 (Reference) |  |  | 1.000 (Reference) |  |
| Q2 | 0.94 (0.82 - 1.07) | 0.351 |  | 0.95 (0.83 - 1.08) | 0.431 |  | 0.95 (0.83 - 1.09) | 0.452 |
| Q3 | 1.03 (0.91 - 1.18) | 0.635 |  | 1.04 (0.92 - 1.19) | 0.516 |  | 1.05 (0.92 - 1.20) | 0.479 |
| Q4 | 1.29 (1.14 - 1.47) | <0.001 |  | 1.32 (1.16 - 1.50) | <0.001 |  | 1.32 (1.15 - 1.52) | <0.001 |
| P for trend | <0.001 |  |  | <0.001 |  |  | <0.001 |  |

Table S4 Baseline characteristics of individuals before and after PSM, excluding individuals over 80 years of age.

| Variable | Before PSM | | | | After PSM | | | |
| --- | --- | --- | --- | --- | --- | --- | --- | --- |
|  | ADL Unlimited  (n = 7600) | ADL  Limited  (n = 1446) | *P* | SMD | ADL-IADL Unlimited  (n = 4988) | ADL-IADL Limited  (n = 1418) | *P* | SMD |
| Age, Mean ± SD | 57.92 ± 8.95 | 61.98 ± 9.05 | <0.001 | 0.449 | 60.77 ± 8.55 | 61.93 ± 9.01 | <0.001 | 0.129 |
| Gender, n (%) |  |  | <0.001 |  |  |  | 0.130 |  |
| female | 3589 (47.22) | 594 (41.08) |  | -0.125 | 2174 (43.58) | 586 (41.33) |  | -0.046 |
| male | 4011 (52.78) | 852 (58.92) |  | 0.125 | 2814 (56.42) | 832 (58.67) |  | 0.046 |
| Drinking, n (%) |  |  | 0.196 |  |  |  | 0.835 |  |
| no | 4635 (60.99) | 908 (62.79) |  | 0.037 | 3105 (62.25) | 887 (62.55) |  | 0.006 |
| yes | 2965 (39.01) | 538 (37.21) |  | -0.037 | 1883 (37.75) | 531 (37.45) |  | -0.006 |
| Smoking, n (%) |  |  | 0.268 |  |  |  | 0.602 |  |
| no | 4623 (60.83) | 902 (62.38) |  | 0.032 | 3068 (61.51) | 883 (62.27) |  | 0.016 |
| yes | 2977 (39.17) | 544 (37.62) |  | -0.032 | 1920 (38.49) | 535 (37.73) |  | -0.016 |
| Hypertension, n (%) |  |  | <0.001 |  |  |  | <0.001 |  |
| no | 5751 (75.67) | 954 (65.98) |  | -0.205 | 3543 (71.03) | 936 (66.01) |  | -0.106 |
| yes | 1849 (24.33) | 492 (34.02) |  | 0.205 | 1445 (28.97) | 482 (33.99) |  | 0.106 |
| Diabetes, n (%) |  |  | <0.001 |  |  |  | 0.004 |  |
| no | 7200 (94.74) | 1307 (90.39) |  | -0.148 | 4632 (92.86) | 1284 (90.55) |  | -0.079 |
| yes | 400 (5.26) | 139 (9.61) |  | 0.148 | 356 (7.14) | 134 (9.45) |  | 0.079 |
| Dyslipidemia, n (%) |  |  | <0.001 |  |  |  | 0.033 |  |
| no | 6795 (90.81) | 1241 (87.33) |  | -0.104 | 4465 (89.51) | 1241 (87.52) |  | -0.060 |
| yes | 688 (9.19) | 180 (12.67) |  | 0.104 | 523 (10.49) | 177 (12.48) |  | 0.060 |
| Marriage, n (%) |  |  | <0.001 |  |  |  | 0.079 |  |
| others | 815 (10.72) | 216 (14.94) |  | 0.118 | 649 (13.01) | 210 (14.81) |  | 0.051 |
| Marriage | 6785 (89.28) | 1230 (85.06) |  | -0.118 | 4339 (86.99) | 1208 (85.19) |  | -0.051 |
| Rural, n (%) |  |  | <0.001 |  |  |  | 0.315 |  |
| Urban | 2733 (35.96) | 403 (27.87) |  | -0.180 | 1465 (29.37) | 397 (28.00) |  | -0.031 |
| Rural, | 4867 (64.04) | 1043 (72.13) |  | 0.180 | 3523 (70.63) | 1021 (72.00) |  | 0.031 |
| Educational, n (%) |  |  | <0.001 |  |  |  | 0.076 |  |
| Primary and below | 3411 (44.92) | 894 (61.83) |  | 0.348 | 2883 (57.80) | 870 (61.35) |  | 0.073 |
| Junior secondary school | 1728 (22.75) | 291 (20.12) |  | -0.066 | 1093 (21.91) | 288 (20.31) |  | -0.040 |
| High school and above | 2455(32.33) | 261 (13.55) |  | -0.235 | 1012 (20.29) | 260 (18.33) |  | -0.061 |

Table S5 Associations of TyG and its related parameters with ADL after propensity score matching, excluding individuals over 80 years of age.

| Variables | Model1 | |  | Model2 | | Model3 | |
| --- | --- | --- | --- | --- | --- | --- | --- |
|  | OR (95%CI) | *P* |  | OR (95%CI) | *P* | OR (95%CI) | *P* |
| TyG | 1.41 (1.30 - 1.54) | <0.001 |  | 1.42 (1.30 - 1.54) | <.001 | 1.395 (1.276 - 1.524) | <0.001 |
| TyG-BMI | 1.04 (1.03 - 1.05) | <0.001 |  | 1.04 (1.03 - 1.06) | <0.001 | 1.04 (1.02 - 1.06) | <0.001 |
| TyG -WC | 1.02 (1.01 - 1.003) | <0.001 |  | 1.02 (1.01 - 1.03) | <0.001 | 1.02 (1.01 - 1.03) | <0.001 |
| TyG -WhtR | 1.22 (1.11 - 1.33) | <0.001 |  | 1.25 (1.14 - 1.38) | <0.001 | 1.24 (1.12 - 1.37) | <0.001 |
| TyG |  |  |  |  |  |  |  |
| Q1 | 1.000 (Reference) |  |  | 1.000 (Reference) |  | 1.000 (Reference) |  |
| Q2 | 1.06 (0.89 - 1.25) | 0.537 |  | 1.04 (0.878 - 1.24) | 0.632 | 1.04 (0.87 - 1.23) | 0.693 |
| Q3 | 0.89(0.76 - 1.07) | 0.233 |  | 0.88 (0.74 - 1.05) | 0.161 | 0.86 (0.72 - 1.03) | 0.103 |
| Q4 | 1.52 (1.29 - 1.79) | <.001 |  | 1.50 (1.28 - 1.77) | <0.001 | 1.44 (1.22 - 1.71) | <0.001 |
| P for trend | <0.001 |  |  | <0.001 |  | <0.001 |  |
| TyG -BMI |  |  |  |  |  |  |  |
| Q1 | 1.000 (Reference) |  |  | 1.000 (Reference) |  | 1.000 (Reference) |  |
| Q2 | 0.94 (0.80 - 1.12) | 0.515 |  | 0.95 (0.80 - 1.13) | 0.593 | 0.95 (0.80 - 1.13) | 0.590 |
| Q3 | 1.04 (0.88 - 1.23) | 0.658 |  | 1.05 (0.89 - 1.25) | 0.551 | 1.05 (0.88 - 1.25) | 0.616 |
| Q4 | 1.39 (1.18 - 1.67) | <0.001 |  | 1.42(1.20 - 1.68) | <0.001 | 1.38 (1.15 - 1.65) | <0.001 |
| P for trend | <0.001 |  |  | <0.001 |  | <0.001 |  |
| TyG -WC |  |  |  |  |  |  |  |
| Q1 | 1.000 (Reference) |  |  | 1.000 (Reference) |  | 1.000 (Reference) |  |
| Q2 | 1.17 (0.99 - 1.40) | 0.073 |  | 1.17 (0.98 - 1.39) | 0.081 | 1.18 (0.99 - 1.40) | 0.072 |
| Q3 | 1.20 (1.08 - 1.43) | 0.040 |  | 1.19 (0.99 - 1.42) | 0.053 | 1.19 (0.99 - 1.43) | 0.053 |
| Q4 | 1.76 (1.49 - 2.08) | <0.001 |  | 1.74 (1.47 - 2.06) | <0.001 | 1.72 (1.44 - 2.05) | <0.001 |
| P for trend | <0.001 |  |  | <0.001 |  | <0.001 |  |
| TyG -WhtR |  |  |  |  |  |  |  |
| Q1 | 1.000 (Reference) |  |  | 1.000 (Reference) |  | 1.000 (Reference) |  |
| Q2 | 0.94(0.79 - 1.11) | 0.439 |  | 0.959 (0.81 - 1.14) | 0.633 | 0.96 (0.81 - 1.14) | 0.654 |
| Q3 | 0.96 (0.81 - 1.13) | 0.609 |  | 0.99 (0.83 - 1.17) | 0.893 | 0.98 (0.83 - 1.17) | 0.840 |
| Q4 | 1.32 (1.12 - 1.55) | <0.001 |  | 1.38 (1.17 - 1.62) | <0.001 | 1.34 (1.12 - 1.60) | 0.001 |
| P for trend | <0.001 |  |  | <0.001 |  | <0.001 |  |

Table S6 Associations of TyG and its related parameters with ADL-IADL after propensity score matching, excluding individuals over 80 years of age.

| Variables | Model1 | |  | Model2 | | Model3 | |
| --- | --- | --- | --- | --- | --- | --- | --- |
|  | OR (95%CI) | *P* |  | OR (95%CI) | *P* | OR (95%CI) | *P* |
| TyG | 1.29 (1.19 - 1.40) | <0.001 |  | 1.29 (1.19 - 1.40) | <.001 | 1.29 (1.19 - 1.41) | <0.001 |
| TyG-BMI | 1.03 (1.02 - 1.04) | <0.001 |  | 1.03 (1.02 - 1.04) | <0.001 | 1.03 (1.02 - 1.05) | <.001 |
| TyG -WC | 1.02 (1.01 - 1.03) | <0.001 |  | 1.02 (1.01 - 1.03) | <0.001 | 1.02 (1.01 - 1.02) | <0.001 |
| TyG -WhtR | 1.19 (1.09 - 1.30) | <0.001 |  | 1.20 (1.10 - 1.31) | <0.001 | 1.21 (1.11 - 1.334 | <0.001 |
| TyG |  |  |  |  |  |  |  |
| Q1 | 1.000 (Reference) |  |  | 1.000 (Reference) |  | 1.000 (Reference) |  |
| Q2 | 1.09 (0.93 - 1.28) | 0.273 |  | 1.09 (0.93 - 1.28) | 0.283 | 1.09 (0.93 - 1.28) | 0.270 |
| Q3 | 1.24 (1.06 - 1.44) | 0.008 |  | 1.23 (1.05 - 1.44) | 0.009 | 1.25 (1.07 - 1.46) | 0.006 |
| Q4 | 1.45 (1.24 - 1.69) | <.001 |  | 1.45 (1.24 - 1.69) | <0.001 | 1.44 (1.229 - 1.693) | <0.001 |
| P for trend | <0.001 |  |  | <0.001 |  | <0.001 |  |
| TyG -BMI |  |  |  |  |  |  |  |
| Q1 | 1.000 (Reference) |  |  | 1.000 (Reference) |  | 1.000 (Reference) |  |
| Q2 | 0.91 (0.78 - 1.07) | 0.251 |  | 0.91 (0.78 - 1.07) | 0.258 | 0.92 (0.78 - 1.07) | 0.275 |
| Q3 | 1.06 (0.91 - 1.23) | 0.472 |  | 1.06 (0.91 - 1.24) | 0.467 | 1.06 (0.91 - 1.25) | 0.444 |
| Q4 | 1.27 (1.09 - 1.47) | 0.002 |  | 1.27 (1.09 - 1.48) | 0.003 | 1.28 (1.09 - 1.51) | 0.003 |
| P for trend | <0.001 |  |  | <0.001 |  | <0.001 |  |
| TyG -WC |  |  |  |  |  |  |  |
| Q1 | 1.000 (Reference) |  |  | 1.000 (Reference) |  | 1.000 (Reference) |  |
| Q2 | 1.13 (0.97 - 1.32) | 0.129 |  | 1.13 (0.96 - 1.32) | 0.135 | 1.13 (0.97 - 1.32) | 0.129 |
| Q3 | 1.06 (0.91 - 1.24) | 0.441 |  | 1.06 (0.91 - 1.24) | 0.473 | 1.07 (0.91 - 1.25) | 0.428 |
| Q4 | 1.38(1.19 - 1.61) | <0.001 |  | 1.38 (1.18 - 1.60) | <.001 | 1.38 (1.17 - 1.62) | <0.001 |
| P for trend | <0.001 |  |  | <0.001 |  | <0.001 |  |
| TyG -WhtR |  |  |  |  |  |  |  |
| Q1 | 1.000 (Reference) |  |  | 1.000 (Reference) |  | 1.000 (Reference) |  |
| Q2 | 0.96 (0.82 - 1.12) | 0.583 |  | 0.96 (0.82 - 1.13) | 0.632 | 0.97 (0.83 - 1.14) | 0.692 |
| Q3 | 1.02 (0.88 - 1.20) | 0.773 |  | 1.03 (0.88 - 1.20) | 0.716 | 1.05 (0.89 - 1.23) | 0.591 |
| Q4 | 1.36 (1.17 - 1.58) | <0.001 |  | 1.37 (1.18 - 1.59) | <0.001 | 1.40 (1.19 - 1.65) | <0.001 |
| P for trend | <0.001 |  |  | <0.001 |  | <0.001 |  |

Table S7 Associations of TyG and its related parameters with ADL, including physical activity and inflammatory markers .

| Variables | Model1 | |  | Model2 | | Model3 | |
| --- | --- | --- | --- | --- | --- | --- | --- |
|  | OR (95%CI) | *P* |  | OR (95%CI) | *P* | OR (95%CI) | *P* |
| TyG | 1.26 (1.19 - 1.33) | <0.001 |  | 1.31 (1.21 - 1.43) | <0.001 | 1.25 (1.14 - 1.37) | <0.001 |
| TyG-BMI | 1.13 (1.07 - 1.19) | <0.001 |  | 1.22 (1.12 - 1.32) | 0.004 | 1.14 (1.04 - 1.25) | 0.005 |
| TyG -WC | 1.25 (1.18 - 1.32) | <0.001 |  | 1.31 (1.20 - 1.43) | <0.001 | 1.25 (1.14 - 1.37) | <0.001 |
| TyG -WhtR | 1.05 (1.00 - 1.11) | 0.066 |  | 1.18 (1.08 - 1.28) | 0.053 | 1.10 (1.01 - 1.21) | 0.040 |

Model 1: Unadjusted .Model 2: age and gender were adjusted; model 3: age, gender, residence,marriage,educationlevel,smoking,drinking,hypertension,dyslipidemia ,diabetes , exercise and white blood cell were adjusted.

Table S8 Associations of TyG and its related parameters with ADL-IADL, including physical activity and inflammatory markers.

| Variables | Model1 | |  | Model2 | | Model3 | |
| --- | --- | --- | --- | --- | --- | --- | --- |
|  | OR (95%CI) | *P* |  | OR (95%CI) | *P* | OR (95%CI) | *P* |
| TyG | 1.20 (1.15 - 1.25) | <0.001 |  | 1.17 (1.09 - 1.26) | <0.001 | 1.18 (1.09 - 1.27) | <0.001 |
| TyG-BMI | 1.13 (1.08 - 1.18) | <0.001 |  | 1.13 (1.05 - 1.21) | 0.004 | 1.14 (1.05 - 1.23) | 0.005 |
| TyG -WC | 1.14 (1.09 - 1.19) | <0.001 |  | 1.14 (1.06 - 1.22) | <0.001 | 1.14 (1.05 - 1.23) | <0.001 |
| TyG -WhtR | 1.11 (1.07 - 1.16) | <0.001 |  | 1.13 (1.06 - 1.21) | <0.001 | 1.14 (1.06 - 1.23) | <0.001 |

Model 1: Unadjusted .Model 2: age and gender were adjusted; model 3: age, gender, residence,marriage,educationlevel,smoking,drinking,hypertension,dyslipidemia ,diabetes , exercise and white blood cell were adjusted.

Table S9 Associations of TyG and its related parameters with ADL, excluding individuals BMI > 40.

| Variables | Model1 | |  | Model2 | | Model3 | |
| --- | --- | --- | --- | --- | --- | --- | --- |
|  | OR (95%CI) | *P* |  | OR (95%CI) | *P* | OR (95%CI) | *P* |
| TyG | 1.20 (1.15 - 1.25) | <0.001 |  | 1.17 (1.09 - 1.26) | <0.001 | 1.18 (1.09 - 1.27) | <0.001 |
| TyG-BMI | 1.13 (1.08 - 1.18) | <0.001 |  | 1.13 (1.05 - 1.21) | 0.004 | 1.14 (1.05 - 1.23) | 0.005 |
| TyG -WC | 1.14 (1.09 - 1.19) | <0.001 |  | 1.14 (1.06 - 1.22) | <0.001 | 1.14 (1.05 - 1.23) | <0.001 |
| TyG -WhtR | 1.11 (1.07 - 1.16) | <0.001 |  | 1.13 (1.06 - 1.21) | <0.001 | 1.14 (1.06 - 1.23) | <0.001 |

Model 1: Unadjusted .Model 2: age and gender were adjusted; model 3: age, gender,residence,marriage,educationlevel,smoking,drinking,hypertension,dyslipidemia and diabetes were adjusted.

Table S10 Associations of TyG and its related parameters with ADL-IADL, excluding individuals BMI > 40.

| Variables | Model1 | |  | Model2 | | Model3 | |
| --- | --- | --- | --- | --- | --- | --- | --- |
|  | OR (95%CI) | *P* |  | OR (95%CI) | *P* | OR (95%CI) | *P* |
| TyG | 1.26(1.19 - 1.33) | <0.001 |  | 1.26 (1.20 - 1.33) | <0.001 | 1.24(1.17 - 1.31) | <0.001 |
| TyG-BMI | 1.14(1.08 - 1.20) | <0.001 |  | 1.18 (1.11 - 1.24) | <0.001 | 1.15(1.09 - 1.22) | <0.001 |
| TyG -WC | 1.25(1.18 - 1.32) | <0.001 |  | 1.25 (1.18 - 1.32) | <0.001 | 1.22(1.16 - 1.30) | <0.001 |
| TyG -WhtR | 1.06(1.01 - 1.12) | 0.042 |  | 1.14 (1.08 - 1.20) | <0.001 | 1.11 (1.05 - 1.18) | <0.001 |

Model 1: Unadjusted .Model 2: age and gender were adjusted; model 3: age, gender,residence,marriage,educationlevel,smoking,drinking,hypertension,dyslipidemia and diabetes were adjusted.
